# Assessment of Pelvic Motion During Single-Leg Weight-Bearing Tasks Using Smartphone Sensors: Validity Study

**DOI:** 10.2196/65342

**Published:** 2025-04-01

**Authors:** Yu Xi, Zhongsheng Li, Surendran Vatatheeswaran, Valter Devecchi, Alessio Gallina

**Affiliations:** 1 School of Sport, Exercise and Rehabilitation Sciences, College of Life Sciences, University of Birmingham, Y14, Birmingham, B15 2TT, United Kingdom, 44 0121 4158187

**Keywords:** smartphone, kinematics, acceleration, exercise, squat

## Abstract

**Background:**

Clinicians and athletic training specialists often assess the performance of single-leg, weight-bearing tasks to monitor rehabilitation progress and guide exercise progression. Some of the key metrics assessed are excessive pelvic motion, balance, and duration of each repetition of the exercise. Motion can be objectively characterized using motion capture (MOCAP); however, MOCAP is often not available in clinics due to the high costs and complexity of the analyses. Smartphones have built-in sensors that can be used to measure changes in body segment orientation and acceleration, which may make them a more feasible and affordable technology to use in practice.

**Objective:**

This study aimed to determine if, compared to gold-standard MOCAP, smartphone sensors can provide valid measures of pelvic orientation, acceleration, and repetition duration during single-leg tasks in healthy individuals.

**Methods:**

Overall, 52 healthy participants performed single-leg squats and step-down tasks from heights of 15 and 20 cm. Pelvic motion was assessed using MOCAP and a smartphone placed over the sacrum. The MATLAB (MathWorks) mobile app was used to collect smartphone acceleration and orientation data. Individual repetitions of each exercise were manually identified, and the following outcomes were extracted: duration of the repetition, mediolateral acceleration, and 3D pelvic orientation at peak squat. Validity was assessed by comparing metrics assessed with a smartphone and MOCAP using intraclass correlation coefficients (ICCs) and paired Wilcoxon tests. Differences between tasks were compared using 1-way ANOVA or the Friedman test.

**Results:**

Across the 3 single-leg tasks, smartphone estimates demonstrated consistently high agreement with the MOCAP for all metrics (ICC point estimates: >0.8 for mediolateral acceleration and frontal plane orientation; >0.9 for squat duration and orientation on the sagittal and transverse plane). Bias was identified for most outcomes (multiple *P*<.001). Both smartphone and MOCAP recordings identified clear differences between tasks, with step-down tasks usually requiring larger changes in pelvic orientation and larger mediolateral sways. Duration did not differ between tasks.

**Conclusions:**

Despite a consistent bias, the smartphone demonstrated good to excellent validity relative to gold-standard MOCAP for most outcomes. This demonstrates that smartphones offer an accessible and affordable tool to objectively characterize pelvic motion during different single-leg weight-bearing tasks in healthy participants. Together with earlier reports of good between-day reliability of similar measures during single-leg squats, our results suggest that smartphone sensors can be used to assess and monitor single-leg task performance. Future studies should investigate whether smartphone sensors can aid in the assessment and treatment of people with musculoskeletal disorders. More user-friendly interfaces and data analysis procedures may also facilitate the implementation of this technology in practice.

## Introduction

The single-leg squat (SLS) and the step-down are 2 functional performance tests often used to identify abnormal movement of the trunk, pelvis, and lower limbs [1,2] and to assess single-leg balance in dynamic tasks [3]. Minimal pelvic tilt and rotation, and small postural sways, are factors required to rate squat performance as “good” [4], whereas excessive motion has been associated with various musculoskeletal conditions. For example, individuals with patellofemoral pain often exhibit increased contralateral pelvic drop during SLS in comparison to healthy participants [1]. People with hip pathologies may present greater anterior pelvic tilt during step-down [5] and larger mediolateral sways than healthy participants during SLS [6]. Gender-specific variations in pelvic kinematics have also been described. Compared to males, females demonstrate larger anterior pelvic tilt and pelvic drop during step-down [5] and greater pelvic rotation toward the weight-bearing limb during SLS [7]. These gender-specific movement patterns have been hypothesized to contribute to an increased incidence of anterior cruciate ligament injuries [8] and patellofemoral pain [9] in females. Assessing and monitoring pelvic orientation and acceleration during functional tasks may assist health care providers to identify injury risk factors, design individualized rehabilitation programs, and track rehabilitation progress over time.

SLS performance has traditionally been assessed visually [4,10,11] or in the laboratory [12-14]. Assessment in the clinic is often performed visually [15], although systematic reviews suggest that the validity of most of the procedures used to score squat performance is insufficient [16], and reliability is only moderate [10]. Additionally, research on a number of different tasks and joints shows that it is challenging for clinicians to visually identify single-plane movement changes smaller than 12 degrees [17], suggesting that small alterations in pelvic movement may not be detected visually. Motion can be assessed objectively by using laboratory techniques. Motion capture (MOCAP) is considered the gold standard to assess joint angles, but it is unsuitable to use in practice due to high costs and the time needed to collect and analyze the data. Inertial measurement units (IMUs) have been demonstrated to be valid and reliable in measuring lower limb kinematics [18,19] but still require equipment often not available outside specialized centers [20]. This lack of availability of sensors outside research laboratories is one of the key barriers to the implementation of objective motion measures in practice.

Contemporary smartphones have sensors that are similar to those embedded in laboratory-grade IMUs and may therefore offer a simple solution to assess body kinematics in practice. Compared to dedicated laboratory equipment, which can be expensive and is often not available in clinical practices, smartphones are readily available to the wider population and therefore have the potential to be used as an inexpensive tool to assess motion objectively. Smartphone sensors are valid and reliable when measuring static joint range of motion [21-23] and when assessing balance [24-26]. In the past, smartphones have been used to assess pelvic orientation in clinical tests [27,28] and during walking [29], as well as acceleration during SLS [30] and sit-to-stand [31]. While smartphone measures of pelvic acceleration and orientation during SLS demonstrated good to excellent reliability between days [32], whether these measures are valid compared to gold-standard MOCAP is yet to be determined.

The objective of this study was to investigate whether smartphone sensors provide valid estimates of pelvic motion during single-leg weight-bearing tasks compared to MOCAP. Specifically, we assessed whether smartphone sensors provide valid estimates of pelvic orientation in the frontal, sagittal, and transverse planes; pelvic mediolateral acceleration; and exercise duration. We further investigated whether smartphone sensors can objectively characterize pelvic motion differences between different single-leg weight-bearing tasks.

## Methods

### Participants

Overall, 52 participants from the University of Birmingham student population (26 females and 26 males, with a mean age of 24.9, SD 4.4 years; weight of 68.1, SD 12.3 kg; and height of 170.7, SD 9.5 cm) were enrolled in the study. The inclusion criteria were a population (1) aged 18‐50 years, (2) with no present lower limb or lower back pain or injuries, (3) with no history of lower limb or lower back surgery, (4) with no conditions that may impair balance or movement, and (5) who are able to communicate in English.

### Ethical Considerations

Ethical approval was obtained from the School of Sport, Exercise Science and Rehabilitation of the University of Birmingham (MCR2223_23). All participants signed an informed consent form before participating in the study and could request to opt out of the study. All data were deidentified before the analysis. The participants did not receive any remuneration but could request that the time spent in the laboratory be counted as research credits.

### Procedures

After a 5-minute, self-directed warm-up, participants performed 3 weight-bearing single-leg tasks (SLS, step-down tasks from 15 cm [SD15], and step-down tasks from 20 cm [SD20]) in a random order using a computer-generated sequence. Since neither pelvic orientation [32] nor balance [33] are affected by leg dominance, tasks were performed on the right leg, which was the preferred leg for kicking a ball in 47 of 52 participants. To better simulate clinical practice, participants wore exercise shoes for the test. Participants performed all tasks with their arms crossed in front of the chests. Participants performed 5 repetitions for each task following a digital metronome set to maintain a tempo of 60 beats per minute; they were instructed to squat down and up in 4 seconds, with a minimum rest interval of 2 seconds between repetitions. Prior to recording, a practice trial was conducted to ensure proper pace and technique. Repetitions where a participant lost balance and placed their foot on the ground were repeated. To easily identify the start and end of each set of squats in the recordings, participants stood quietly for 5 seconds before the task.

SLSs were performed as follows. To standardize the squat depth, a white T-shaped tape measuring 3×10 cm horizontally and 2×10 cm vertically was applied to the floor [34]. Participants positioned their right foot along the T-shaped marker’s long axis, with the tip of their toe just before the marker’s short axis. The participants’ left thigh was vertical, with the knee bent to 90 degrees [34]. Participants were asked to squat down, bringing the knee of the supporting leg forward until the tape in front of their toes disappeared from view. This procedure was followed to aim for a knee flexion of approximately 60 degrees, which is a knee flexion angle usually considered appropriate when performing SLSs [4,13].

Participants performed the step-down from 2 block heights: 15 and 20 cm. The block heights were selected to align with the step height typically used in clinical practice [35]. For each step-down exercise, participants positioned both feet on a block. From the beginning position, participants shifted their weight onto their right leg, extended their left leg anteriorly, and squatted down until their heel lightly touched the floor [36]. Participants who could not reach the floor were instructed to squat down as much as possible while maintaining balance. A researcher took note of when this happened.

### Data Collection

**Figure 1.**
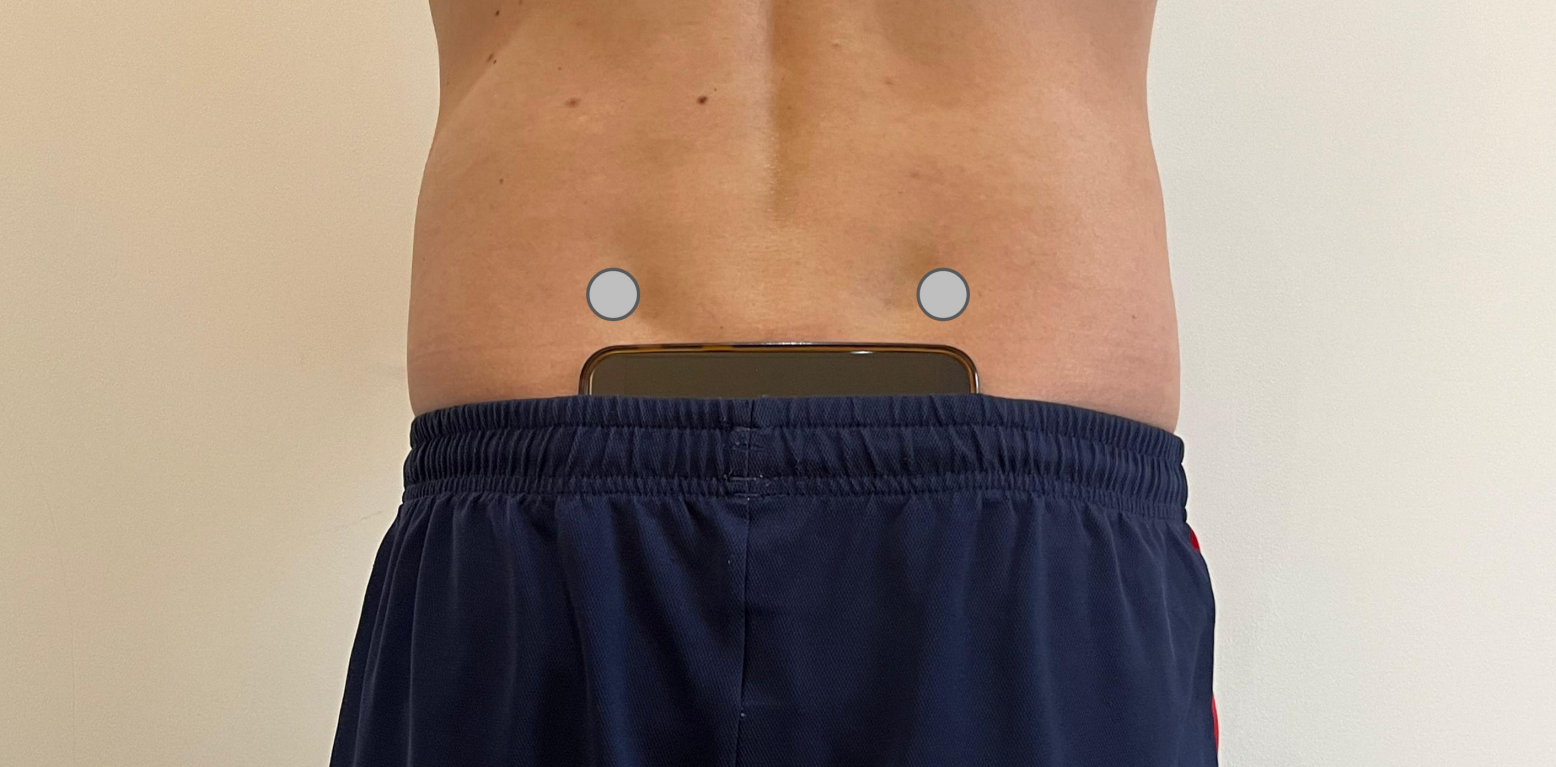
Placement of the smartphone and of the motion capture markers on the posterior superior iliac spines (grey circles).

Before starting data collection, the maximal knee flexion angle during SLS, SD15, and SD20 was measured using a goniometer while participants held a static squat position for each task. During all tasks, pelvic kinematics was simultaneously collected using MOCAP and a smartphone. The MOCAP (8 cameras, Gaitlab, BTS Bioengineering; sampling rate 250 Hz) tracked the position of 4 reflective markers (12 mm diameter) placed on the participants’ bilateral anterior superior iliac spines and posterior superior iliac spines (Figure 1). A smartphone (Android, Samsung A5) was placed in landscape orientation on the participants’ sacrum, with the screen oriented outward and the camera to the left. We chose to place the smartphone over the sacrum in accordance with a prior study that demonstrated good or excellent between-day reliability [32]. The MATLAB (MathWorks) mobile app was used to collect smartphone orientation and acceleration at 100 Hz; smartphone orientation data were estimated from rotation vectors collected by the smartphone’s virtual orientation sensor, which integrates accelerometer, gyroscope, and magnetometer data. To ensure consistent placement throughout the tasks without the need for additional equipment, the smartphone was secured in place using the elastic band of the participant’s trousers at the waist.

### Data Analysis

**Figure 2.**
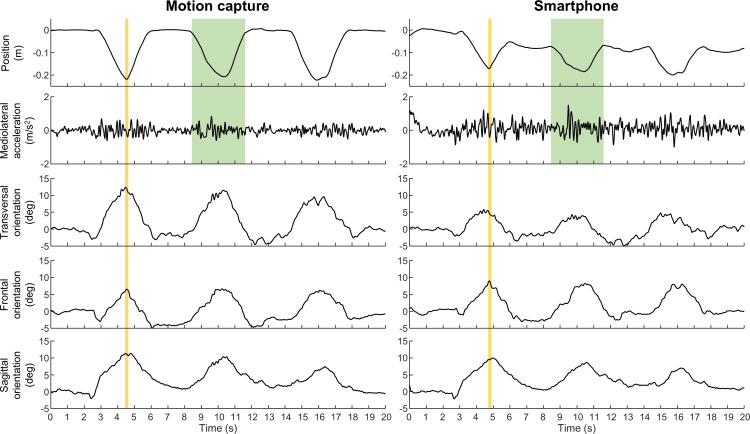
Example of the vertical position waveform, mediolateral acceleration, and pelvic orientation in the transverse, frontal, and sagittal planes during 3 cycles of a step-down task (20 cm) measured with motion capture (left) and smartphone (right). Positive values indicate forward contralateral pelvic rotation, contralateral pelvic drop, and anterior pelvic tilt, respectively. The yellow shaded areas indicate the 200-millisecond window centered on the peak squat, used to analyze pelvic orientation. The green shaded areas depict the window from the start to the end of the second squat, used to analyze squat duration and mediolateral acceleration.

The data were analyzed using MATLAB and Microsoft Excel. For the MOCAP data, pelvic orientation was calculated from the marker position using trigonometry; the average position of the 4 markers was used to calculate vertical pelvic displacement and mediolateral pelvic displacement, which was double-differentiated to obtain mediolateral pelvic acceleration. The smartphone orientation was collected as roll (sagittal plane), pitch (frontal plane), and azimuth (transverse plane). Both MOCAP and smartphone orientation data were adjusted so that larger values indicate greater anterior pelvic tilt, contralateral pelvic drop, and forward contralateral pelvic rotation. The smartphone acceleration includes data in the X, Y, and Z axes, representing vertical, mediolateral, and anteroposterior directions, respectively. An approximation of the vertical displacement of the smartphone was calculated by double integration of the vertical acceleration signal, applying a high-pass filter (2nd order Butterworth, 0.1 Hz) at each step to reduce the drift. All data were filtered before analysis (4th order Butterworth; 10 Hz low-pass filter for orientation data; 1 Hz high-pass filter for acceleration data). These data were exported to Microsoft Excel to manually identify the start, end, and peak of each squat, as well as a stable baseline value. These time indices were identified visually from plots, separately for the MOCAP and smartphone vertical displacement. The baseline was identified as a time instant in 5 seconds before the start of the first repetition with stable pelvic orientation. The start and end of the movement were identified as the points where the pelvic vertical displacement deviated from the baseline. Peak squat was identified as the lowest pelvic vertical displacement, or the middle point of the pelvic vertical displacement plateau if the participant paused when they reached the maximal squat depth. Visual identification of these values was determined to be more appropriate than a programmatic approach because of distortions of the smartphone vertical displacement data due to the double integration of acceleration data and differences in how participants performed the task. Pelvic orientation at peak squat was calculated as the average orientation in the frontal, sagittal, and transversal planes in a 200-millisecond window centered on the identified peak squat (Figure 2). For each task and repetition, the pelvic orientation in the 3 planes was calculated by subtracting the baseline from the pelvic orientation measurements at the peak squat. The squat duration was determined as the difference between the start and end of the movement. The mediolateral acceleration was quantified as the standard deviation of the mediolateral acceleration signal between the start and end of each repetition. For each outcome and task, the median value across the 5 repetitions was used for the analysis.

### Statistics

SPSS (version 27, IBM) was used for statistical analyses. The presence of systematic bias between MOCAP and smartphone measures was calculated using a 2-tailed, paired sample *t* test for each outcome measure. When the Shapiro-Wilk test identified significant deviations from normal distributions, the Wilcoxon signed-rank test was used instead. The level of agreement between the 2 measurements was evaluated using the intraclass correlation coefficient (ICC) and Bland-Altman plots. ICC estimates and 95% CI were calculated using 2-way mixed-effects models for average measures; consistency or absolute agreement models were used depending on whether there was a significant difference between MOCAP and smartphone data or not. The ICC levels were categorized as follows based on the lower bound of the confidence interval of the ICC: ＜0.5 (poor), 0.5‐0.75 (moderate), 0.75‐0.9 (good), and＞0.9 (excellent) [37].

Repeated measured ANOVA (or Friedman tests for variables not normally distributed) were used to identify differences in maximum knee flexion (analog goniometer data), pelvic orientation, mediolateral acceleration, and squat duration across tasks, both on smartphone and MOCAP data. When significant differences were identified, pairwise post hoc tests with Bonferroni correction were conducted to decompose the main effects. Gender differences were investigated on the smartphone data using a 2-tailed, independent sample *t* test (or Wilcoxon rank-sum test when not normally distributed).

The significance level was set at *P*<.05, and *P* values are reported after Bonferroni correction when applied. Since most variables exhibited nonnormal distributions, data are generally reported as median (IQR).

## Results

During SD15 and SD20, 11 and 19 participants, respectively, were unable to reach the floor. Smartphone data from one participant demonstrated visible drift when measuring orientation in the transverse plane during SD20; this participant was excluded from analyses of this task.

**Figure 3.**
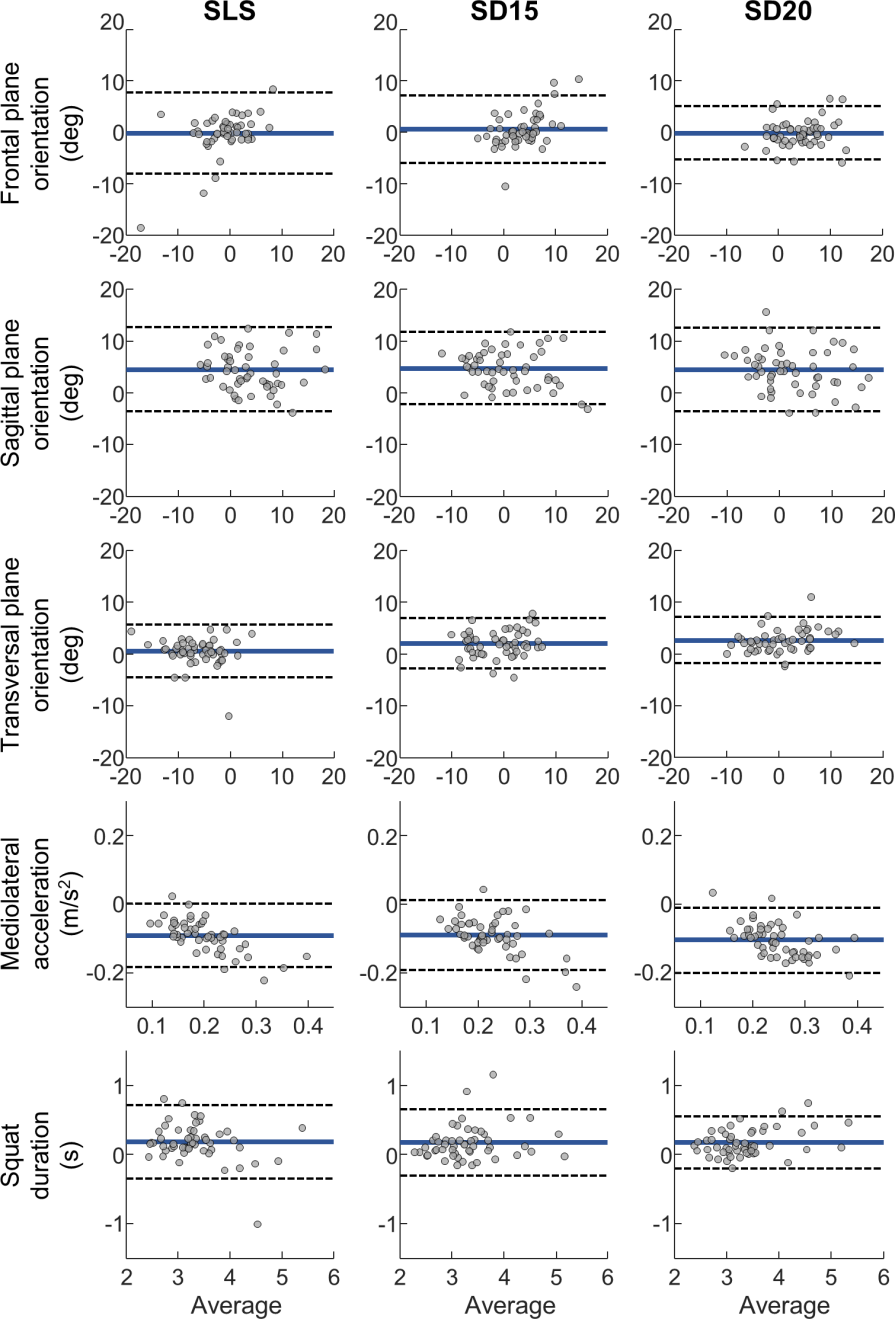
Bland-Altman plots comparing variables measured using motion capture and smartphone during single-leg squat (SLS) and step-down tasks from 15 cm (SD15) or 20 cm (SD20). The thick black line represents the mean difference between motion capture and smartphone. The dashed lines illustrate the 95% limits of agreement.

**Figure 4.**
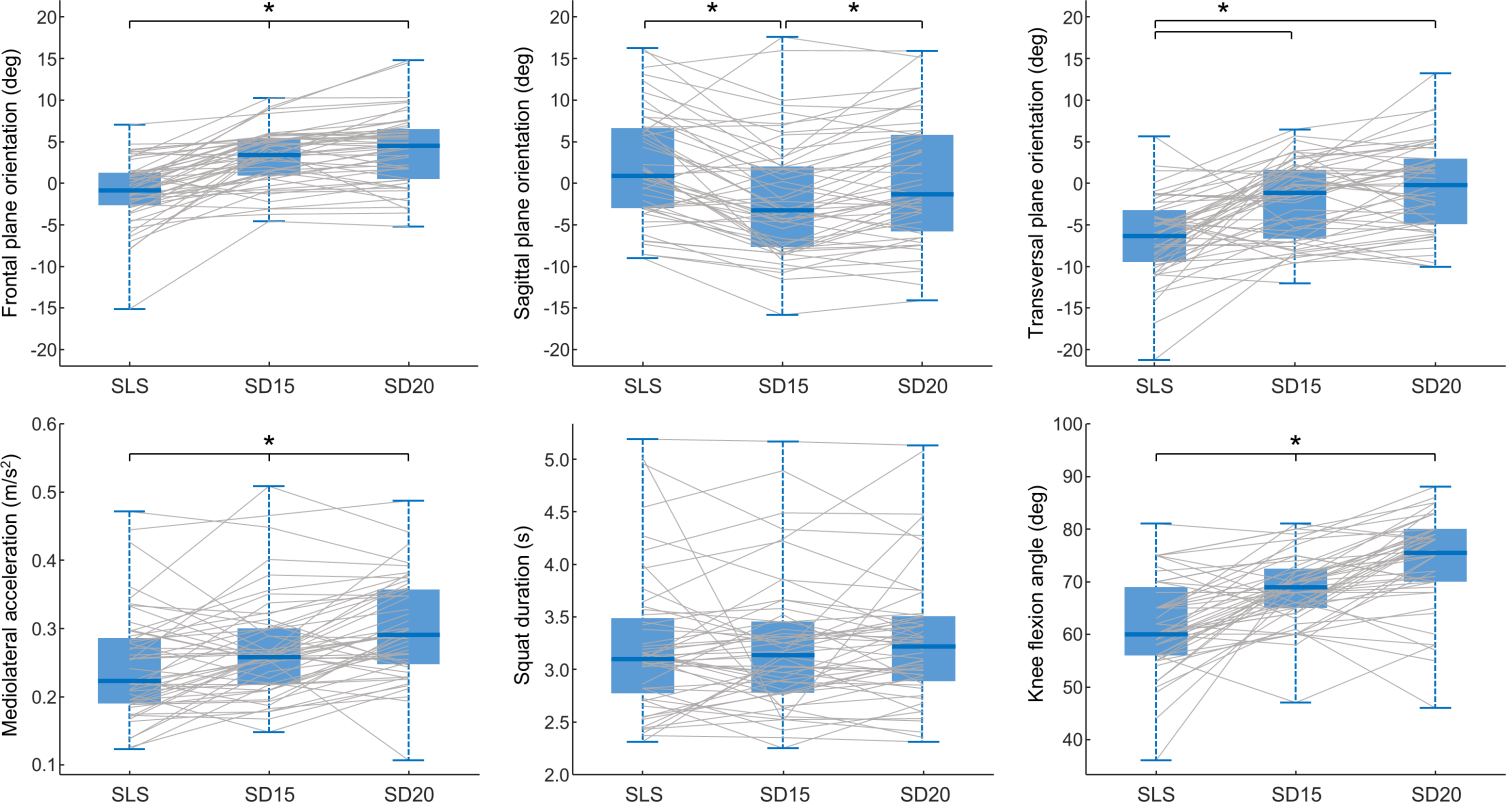
Boxplots comparing pelvic orientation, mediolateral acceleration, squat duration, and knee flexion angle. In the transverse, frontal, and sagittal orientations, positive values indicate forward contralateral pelvic rotation, contralateral pelvic drop, and anterior pelvic tilt. Each boxplot represents data during single-leg squat (SLS) and step-down tasks from 15 cm (SD15) and 20 cm (SD20) steps, and the grey lines are individual participant data. *P<.05.

Group values, bias analysis, ICCs, and between-gender comparisons are presented in Table 1. Bland-Altman plots comparing measurements by MOCAP and the smartphone are shown in Figure 3. Kinematic differences between tasks are presented in Table 2 and graphically as boxplots in Figure 4.

**Table 1.** Validity of smartphone data compared to motion capture. This table presents estimates (median and IQR) of pelvic orientation, mediolateral acceleration, and squat duration. Smartphone data are also presented by gender. Results of the validity analysis are presented as intraclass correlation coefficient (ICC) with 95% CIs to describe agreement and *P* values for the bias analysis. Statistics of the between-group analyses are also presented. Significant comparisons (*P*<.05) are highlighted in italics.

	Motion capture, median (IQR)	Smartphone, median (IQR)	ICC^a^ (95% CI)	Bias (*P* value)	Gender differences		
					Smartphone, median (IQR)		*P* value
					Females	Males	
Pelvic orientation (deg)							
SLS^b^ transversal	–6.2 (–8.9 to –3.1)	–6.4 (–9.5 to –3.3)	0.92 (0.86‐0.95)	.13	–7.5 (–11.0 to –4.0)	–5.6 (–8.3 to –2.5)	.05
SLS frontal	–0.6 (–3.9 to 2.4)	–0.9 (–2.6 to 1.2)	0.81 (0.66‐0.89)	.32	–1.5 (–2.9 to 0.9)	–0.3 (–1.6 to 1.4)	.31
SLS sagittal	5.2 (2.3 to 8.9)	0.9 (–3.0 to 6.5)	0.90 (0.82‐0.94)	<.001	1.2 (–3.0 to 6.7)	0.9 (–3.0 to 6.2)	.39
SD15^c^ transversal	0.8 (–4.6 to 3.9)	–1.2 (–6.5 to 1.5)	0.93 (0.88‐0.96)	<.001	–3.7 (–7.5 to 0.6)	0.2 (–3.5 to 1.9)	.07
SD15 frontal	3.8 (0.2 to 6.2)	3.4 (0.9 to 5.3)	0.83 (0.70‐0.90)	.24	3.5 (1.4 to 5.4)	3.3 (0.5 to 5.2)	.86
SD15 sagittal	1.8 (–2.2 to 7.3)	–3.2 (–7.6 to 2.0)	0.92 (0.87‐0.96)	<.001	–0.2 (–4.5 to 5.1)	–4.5 (–7.8 to –0.2)	.05
SD20^d^ transversal	1.5 (–2.3 to 6.5)	–0.2 (–4.9 to 2.9)	0.96 (0.93‐0.98)	<.001	–0.3 (–4.0 to 5.5)	2.9 (–0.7 to 7.1)	.08
SD20 frontal	3.8 (0.3 to 6.9)	4.5 (0.5 to 6.4)	0.91 (0.84‐0.95)	.68	4.4 (–0.3 to 6.1)	4.9 (2.2 to 7.2)	.28
SD20 sagittal	4.1 (–0.7 to 9.3)	–1.3 (–5.7 to 5.7)	0.91 (0.84‐0.95)	<.001	–1.7 (–5.0 to 8.2)	–1.2 (–5.7 to 3.9)	.39
Mediolateral acceleration (m/s^2^)							
SLS	0.15 (0.12 to 0.18)	0.22 (0.19 to 0.28)	0.84 (0.72‐0.91)	<.001	0.22 (0.19 to 0.29)	0.24 (0.19 to 0.28)	.85
SD15	0.17 (0.14 to 0.21)	0.26 (0.22 to 0.30)	0.80 (0.65‐0.88)	<.001	0.26 (0.22 to 0.29)	0.26 (0.23 to 0.33)	.38
SD20	0.19 (0.16 to 0.22)	0.29 (0.25 to 0.36)	0.82 (0.68‐0.89)	<.001	0.26 (0.23 to 0.36)	0.30 (0.28 to 0.35)	.14
Duration (seconds)							
SLS	3.4 (3.0 to 3.7)	3.1 (2.8 to 3.5)	0.95 (0.91‐0.97)	<.001	2.9 (2.5 to 3.2)	3.1 (3.1 to 3.7)	.01
SD15	3.3 (3.0 to 3.7)	3.1 (2.8 to 3.5)	0.96 (0.93‐0.98)	<.001	3.0 (2.8 to 3.3)	3.2 (2.9 to 3.6)	.11
SD20	3.3 (3.0 to 3.6)	3.2 (2.9 to 3.5)	0.98 (0.96‐0.99)	<.001	3.0 (2.7 to 3.4)	3.3 (3.0 to 3.7)	.04

a ICC: intraclass correlation coefficient.

b SLS: single-leg squat.

c SD15: step-down tasks from 15 cm.

d SD20: step-down tasks from 20 cm.

**Table 2.** Comparison of pelvic orientation, mediolateral acceleration, and duration between tasks. Significant comparisons (*P*<.05) are highlighted in italics.

	Motion capture (*P* value)		Smartphone (*P* value)	
Outcome	Overall test	Post hoc	Overall test	Post hoc
Pelvic orientation				
Transverse (deg)	<.001		<.001	
SLS^a^<SD15^b^		<.001		<.001
SLS<SD20^c^		<.001		<.001
SD15<SD20		.003		—^d^
SD15=SD20		—		.055
Frontal (deg)	<.001		<.001	
SLS<SD15		<.001		<.001
SLS<SD20		<.001		<.001
SD15=SD20		.44		—
SD15<SD20		—		.003
Sagittal (deg)	.002		.001	
SLS>SD15		<.001		<.001
SLS=SD20		.09		.10
SD15<SD20		<.001		<.001
Mediolateral acceleration (m/s^2^)	<.001		<.001	
SLS<SD15		<.001		.02
SLS<SD20		<.001		<.001
SD15<SD20		.02		<.001
Duration (seconds)	.63	—	.76	—

a SLS: single-leg squat.

b SD15: step-down tasks from 15 cm.

c SD20: step-down tasks from 20 cm.

d Not applicable.

A systematic bias between MOCAP and smartphone data was observed in 5 pelvic orientation variables (SLS sagittal, SD15 sagittal, SD15 transverse, SD20 sagittal, and SD20 transverse), all acceleration variables, and all duration variables. In comparison to MOCAP, the smartphone tended to measure consistently lower anterior pelvic tilt across all tasks (SLS: median 4.1 [IQR 1.8-7] deg, *z*=−5.71, *P*<.001; SD15: median 4.5 [IQR 2.2-7.3] deg, *t*_51_=9.51, *P*<.001; SD20: median 4.3 [IQR 2-7.3] deg, *z*=−5.53, *P*<.001). In the transverse plane, the smartphone measured less contralateral pelvic anterior rotation than MOCAP during SD15 (median 1.9 [IQR 0.4-3.8] deg, *z*=−4.85, *P*<.001) and SD20 (median 2.4 [IQR 1.1-3.7] deg, *t*_50_=8.27, *P*<.001). For mediolateral acceleration, the smartphone accelerometer recorded approximately 0.1 m/s^2^ larger acceleration than MOCAP for all tasks (SLS: median −0.09 [IQR −0.11 to −0.06] m/s^2^, *z*=6.21, *P*<.001; SD15: median −0.09 [IQR −0.11 to −0.06] m/s^2^, *z*=6.22, *P*<.001; SD20: median −0.1 [IQR −0.14 to −0.07] m/s^2^, *z*=6.25, *P*<.001); the Bland-Altman plots demonstrate a proportional bias, with larger bias present for higher accelerations recorded. The smartphone-identified squat durations were approximately 150 milliseconds shorter than those recorded by MOCAP across all tasks (SLS: median 0.17 [IQR 0.08-0.34] seconds, *z*=−4.7, *P*<.001; SD15: median 0.12 [IQR 0.04-0.23] seconds, *z*=−4.48, *P*<.001; SD20: median 0.12 [IQR 0.05-0.32] seconds, *z*=−5.05, *P*<.001).

Regarding the agreement of the measurements obtained from the smartphone and MOCAP, the lower bound of the CI was higher than 0.75 for 7 out of 9 orientation outcomes, which indicates at least “good” agreement between the 2 devices in assessing pelvic orientation during SLS, SD15, and SD20. Agreement of mediolateral acceleration demonstrated ICCs with point estimates higher than 0.8, but the lower bound of the CI was between 0.65 and 0.72, and therefore in the “moderate” agreement range. For squat duration, the smartphone demonstrated a high correlation with the MOCAP measurement, with the lower bound of the ICC confidence interval ranging from 0.91 to 0.99, indicating “excellent” agreement between the smartphone and MOCAP.

There were trends for females to demonstrate less contralateral pelvic anterior rotation during SLS (*t*_50_=2.02, *P*=.05) and greater anterior pelvic tilt during SD15 (*t*_50_=−2.01, *P*=.05) compared to males. Squat duration was longer for males than females during both SLS (*z*=−2.48, *P*=.01) and SD20 (*z*=−2.08, *P*=.04), while no such difference was noted for SD15 (*z*=−1.62, *P*=.11).

Knee flexion measured with the goniometer differed between tasks (*χ*^2^_2_=48.5, *P*<.001), with increasing knee flexion angles from SLS to SD20 (all comparisons *P*<.001; SLS: median 60 [IQR 56-68.5] deg; SD15: median 69 [IQR 65-72.25] deg; SD20: median 75.5 [IQR 70-80] deg). Smartphone sensors detected significant differences in pelvic kinematics between tasks. In the transverse plane (*χ*^2^_2_=31.8, *P*<.001), participants demonstrated less contralateral anterior rotation during SLS compared to SD20 (median 6.3 [IQR 3.5-9.2] deg, *P*<.001) and SD15 (median 5 [IQR −0.2 to 8.2] deg, *P*<.001). In the frontal plane (*χ*^2^_2_=61.2, *P*<.001), participants demonstrated more contralateral pelvic drop during SD20 than both SLS (median 4.7 [IQR 2.5-7.1] deg, *P*<.001) and SD15 (median 0.9 [IQR 0-2.2] deg, *P*=.003), and during SD15 compared to SLS (median 3.9 [IQR 1.6-6.3] deg, *P*<.001). In the sagittal plane (*F*_1.66,84.86_=15.77, *P*<.001), the pelvis was in less anterior pelvic tilt during SD15 than both SLS (median −4.3 [IQR −6.3 to −0.4] deg, *P*<.001) and SD20 (median −1.7 [IQR −4.4 to 0.6] deg, *P*<.001). Mediolateral acceleration differed between tasks (*χ*^2^_2_=30.7, *P*<.001), with lower values during SLS than both SD15 (median −0.02 [IQR −0.06 to 0.01] m/s^2^, *P*=.015) and SD20 (median −0.06 [IQR −0.10 to −0.03] m/s^2^, *P*<.001), and during SD15 compared to SD20 (median −0.03 [IQR −0.06 to 0] m/s^2^, *P*<.001). Squat duration did not differ across tasks (*χ*^2^_2_=0.5, *P*=.76). Overall, MOCAP revealed differences between tasks comparable to those observed using smartphones (Table 2), the main notable exception being a lack of differences in pelvic orientation in the frontal plane when comparing SD15 and SD20 (*P*=.44).

## Discussion

### Main Findings

We observed a high level of agreement between pelvic kinematics measured with a smartphone and MOCAP across tasks, especially for task duration, and orientation on the sagittal and transverse planes. Significant bias was identified for most outcomes. Smartphone sensors detected differences in pelvic motion between weight-bearing tasks, and these differences were generally consistent with those identified using MOCAP. Our findings suggest that smartphone sensors can effectively characterize pelvic kinematics, pelvic acceleration, and exercise duration during single-leg weight-bearing tasks in healthy individuals, which demonstrates their potential to be used as a low-cost technology to assess motion objectively in rehabilitation without the need for specialized equipment.

### Validity of Smartphone Compared to MOCAP

The good validity of smartphone measurement is consistent with previous studies. Several studies have proved the validity of smartphones in assessing body segment orientation during static tasks [21,22]. Jung et al [28] validated a smartphone-based app for measuring pelvic rotation in the transverse plane during single-leg lifting and reported an excellent correlation (ICC=0.99) between the smartphone and MOCAP. However, the pelvic range of motion was assessed in the supine position, which was a task less functional than those assessed in our study. Mizuno et al [29] explored the validity of smartphones in measuring dynamic pelvic orientation in frontal and sagittal planes during walking and found that smartphones provide accurate measurement of pelvic orientation, especially at slower walking speeds, although the error between the smartphone and MOCAP measurements tended to increase with walking speed [29], suggesting an effect of movement speed on the validity of smartphone-recorded data. Compared to the sagittal and transverse planes, orientation in the frontal plane demonstrated lower validity across tasks. This could be possibly explained by a lower variance across participants, which may have made measures on the frontal plane more susceptible to small movements of the smartphone relative to the skin. A good validity of smartphones in assessing balance-related parameters has also been proved in prior studies [24]. Marshall et al [30] observed significant large correlations between smartphone-recorded acceleration data and corresponding force plate measurements (*r*=0.56 and *r*=0.71) when assessing postural sway during squat movements in individuals with and without hip pain. The higher correlation we observed could be explained by the use of pelvic acceleration derived from the MOCAP, as opposed to force plates. The duration of each squat detected from the smartphone waveform in our study showed good agreement with the MOCAP measurement, with ICCs ranging from 0.91 to 0.99. This was consistent with previous studies that found smartphones to provide valid measurement of step duration during walking [38,39]. It should be noted that measurement validity is highly dependent on the specific characteristics of the task. Our data suggest that smartphone sensors provide valid pelvic orientation and exercise duration data during single-leg weight-bearing exercises; whether the estimates are similarly valid in other conditions, such as highly multiplanar motion or high-speed movements, remains to be determined.

Smartphone sensors measured consistently less anterior pelvic tilt and forward contralateral pelvic rotation, larger acceleration, and shorter squat duration than MOCAP. For pelvic orientation measurements, a consistent bias was identified in the sagittal and transverse planes. Our study observed higher biases and wider limits of agreement in the sagittal plane, but similar biases and limits of agreement in the transverse plane, when compared to IMU used to measure lower-limb joint kinematics during SLS [40]. Smartphone sensors also tended to systematically overestimate the mediolateral acceleration by approximately 0.1 m/s^2^ on average; trends in the Bland-Altman plots show that this difference is minimal when acceleration is low, and especially pronounced at higher accelerations. A systematic overestimation was also observed in a previous study [41], which found that trunk-mounted accelerometers overestimated peak accelerations by 0.85 g compared to MOCAP during locomotion. One possible reason for the overestimation might be that the smartphone records additional linear acceleration besides mediolateral motion, for instance, due to incomplete removal of acceleration due to gravity, leading to higher smartphone acceleration estimates. Bland-Altman analyses indicated that smartphones consistently underestimated squat duration by approximately 150 milliseconds, differently from previous research reporting negligible bias when smartphone accelerometers are used to quantify gait temporal parameters [38,39]. Sources of bias could include hardware and software differences, imprecise smartphone sampling rates, or position and attachment of the smartphone compared to MOCAP; however, we are unable to clearly identify the source of bias from our dataset. Overall, our data demonstrate good agreement and significant, small bias between orientation and acceleration measures collected using smartphone sensors and MOCAP, demonstrating good validity when estimating pelvic kinematics during single-leg weight-bearing tasks.

### Kinematic Differences Between Tasks

We observed consistent differences in pelvic kinematics and acceleration between the single-leg weight-bearing tasks, and these differences were usually comparable when assessed with MOCAP or a smartphone. As expected, the amount of knee flexion was lower for SLS than for the step-down tasks, and for SD15 compared to SD20. Compared to quiet standing, the pelvis remained horizontal and rotated posteriorly during SLS, while during step-down tasks a consistent contralateral pelvic drop and negligible rotation were observed. Similar movement patterns were also described previously when comparing similar tasks [36]. Kinematics during SLS differed from our previous work using smartphones, where we identified minimal pelvic drop and rotation, and larger anterior pelvic tilt [32]. These different movement patterns are likely influenced by different requirements of the tasks. In the SLS performed in this study, the goal was to bring the supporting knee forward to cover the tape while keeping the nonsupporting leg off the floor, whereas SLS performed at home [32] were standardized by asking participants to lightly touch an object placed behind them. The aim of the step-down tasks was to lightly touch the ground with the heel, which could be achieved by increasing contralateral pelvic drop and forward rotation. Pelvic kinematics in the sagittal plane is variable across studies, likely because of differences in the position of the nonsupporting leg [13].

Increasing the step height resulted in greater contralateral pelvic drop and anterior tilt, although differences were small (approximately 2 degrees in the sagittal plane and 1 degree in the frontal plane). Our result agreed with a previous study [36]. However, it is noteworthy that alterations in pelvic kinematics may be related to specific phases of movement. In Lewis et al [36] study, significant differences in pelvic kinematics were only observed at peak knee flexion, with no significant differences at 60 deg of knee flexion. In our study, participants exhibited a maximal knee flexion higher than the 60 deg threshold in 83.3% (130/156) of the tasks. The pronounced changes in pelvic orientation observed during the terminal phase of the step-down tasks suggest that changes in pelvic orientation during the terminal phase of the step-down test may play a crucial role in maintaining balance and biomechanical efficiency.

Mediolateral pelvic acceleration was lower during SLS compared to the step-down tasks and during SD15 compared to SD20. A larger acceleration suggests that step-down tasks challenge balance and smoothness of movement more than SLS, more so for larger step-down heights. This finding aligns with previous research that investigated the biomechanical differences between tasks, since step-down tasks were found to require greater knee flexion, hip flexion, and adduction compared to SLS [36], and therefore likely more challenging for balance.

No significant differences between tasks were observed for squat duration; however, it is notable that the vast majority of the participants completed the task in less than the 4-second duration prescribed by the metronome. Since we only monitored pelvic displacement, it is possible that other joints may have initiated motion before the pelvis, or stopped after the pelvis, leading to an underestimation of the squat duration. However, since one of the main aims of these exercises is to activate the leg muscles, whose activation increases proportionally with squat depth [42-44], monitoring exercise duration as pelvic displacement is likely more relevant than monitoring other joints. Overall, the shorter squat duration suggests that the time under tension of the leg muscles was less than that prescribed (4 seconds). Our results are similar to what was observed using other sensors to measure time under tension during knee exercises [45] and highlight the need to objectively measure exercise dosage in clinical trials and in practice. Given the good validity observed in this study, smartphone sensors may be a possible solution to provide accurate estimates of exercise duration remotely.

### Gender Differences

Minimal gender differences in pelvic orientation were observed across the transverse and sagittal planes during SLS and SD15, while no significant gender differences were identified in the frontal plane. Our results are inconsistent with previous studies that reported significant gender differences in transverse [7,46], sagittal, and frontal planes [5,47]. Specifically, we found females to exhibit less forward contralateral pelvic rotation compared to males during SLS. This contrasts with the findings of Weeks et al [46] and Graci et al [7], who reported females demonstrating greater forward contralateral pelvic rotation, and Zawadka et al [47], who found females to have greater anterior pelvic tilt and contralateral pelvic drop. Moreover, in our study, a significant gender difference was noted in the anterior pelvic tilt during SD15, whereas Lewis et al [5] also found females to demonstrate greater contralateral pelvic drop during the step-down test. Additionally, we found no significant gender differences in mediolateral acceleration across all tasks, which also contrasts with a previous study [48]. The differences in results may be due to variations in task requirements. It has been found that greater squat depth is linked with greater pelvic motion [49], and previous studies that identified differences between genders required a knee flexion of 75‐85 degrees [46] or maximal squat depth [7,47]. In contrast, this study targeted a squat depth of 60 degrees, which is in line with clinical recommendations [4] but is less than previous studies. Our study observed a significant gender difference in squat duration during SLS and SD20, indicating that females had a shorter squat duration than males. The result was similar to a previous study [7], which also found that females performed SLS in less time than males. Since slower squats generate greater muscle tension on lower limb joints than fast squats [50], our results suggest that monitoring exercise dosage may be especially important for females.

### Practical Applications

Our findings support the validity of smartphone sensors to measure pelvic kinematics during single-leg weight-bearing tasks. This adds to evidence that smartphone measures of pelvic kinematics during SLSs are reliable between days when people test themselves at home [32]. Taken together, this evidence suggests that smartphone sensors may be useful to characterize and monitor pelvic kinematics, pelvic acceleration, and squat duration during single-leg weight-bearing tasks over time, which has potential implications for the assessment [4] and treatment [51] of people at risk of lower limb disorders or with musculoskeletal disorders (if validated on that population). This motion analysis could be performed in clinical settings, reducing the reliance on specialized equipment, or remotely, making health care more accessible, particularly in remote areas. Future work may include the simplification of the data analysis process (see Limitations), the definition of a standardized protocol that includes smartphone positioning, patient instructions, and data collection and analysis procedures to ensure consistent data quality.

The kinematic differences observed between different single-leg weight-bearing tasks may be helpful to guide exercise prescription. The specific kinematic and kinetic differences between SLS, SD15, and SD20 observed in this study may help practitioners guide their exercise prescription, depending on the specific target of their treatment. Future studies may investigate if smartphone sensors can assist in identifying kinematic criteria for progression between exercises.

### Limitations

Our study has several limitations. First, due to variations in waistband elasticity among trousers, in some participants the smartphone might have shifted position during the performance of the exercise. While our placement was simple, did not require additional equipment, and resulted in ICC point estimates higher than 0.8, the use of a dedicated elastic band may further increase the validity of smartphone measurements. This may be especially necessary if faster movements are required. Second, the smartphone app we used requires to export the data and analyze it offline; while most of the analysis was performed on Microsoft Excel, which is widely available and requires no coding knowledge, some parts of the process (eg, estimation of displacement from smartphone acceleration) were implemented using a proprietary coding software because they are difficult to implement in spreadsheet editors. A more user-friendly function that displays data waveforms and allows to analyze data on the screen would facilitate implementation in practice. We chose to let participants perform a self-directed warm-up; while this was intended to reflect the variety of settings the procedure may be used in, interindividual differences in warm-up exercises may have contributed to the wide range of kinematic values observed in the study. Lastly, our study only investigated healthy participants who were university students in a rather narrow age range. To generalize our findings, further research should encompass a more diverse range of populations, such as athletes, older people, or people with lower limb pathologies.

### Conclusions

Compared to MOCAP, smartphone sensors provide valid measurements of pelvic motion (orientation, acceleration, and task duration) during single-leg weight-bearing tasks in healthy adults. This can assist health care and sport practitioners in characterizing pelvic motion, balance, and exercise duration in practice, which could be helpful to guide assessment and exercise prescription.

## Abbreviations

ICC: intraclass correlation coefficient
IMU: inertial measurement unit
MOCAP: motion capture
SD15: step-down tasks from 15 cm
SD20: step-down tasks from 20 cm
SLS: single-leg squat
